# Research on the Influence of Environmental Regulation on Social Employment—An Empirical Analysis Based on the STR Model

**DOI:** 10.3390/ijerph17020622

**Published:** 2020-01-18

**Authors:** Xiaohua Wang, Qing Yang, Ning He

**Affiliations:** 1 School of Safety Science and Emergency Management, Wuhan University of Technology, Wuhan 430070, China; wxh3707@whut.edu.cn (X.W.); yangq@whut.edu.cn (Q.Y.); 2 School of Economics and Trade, Henan University of Technology, Zhengzhou 450001, China

**Keywords:** environmental regulation, employment level, STR model

## Abstract

Environmental regulation will affect social employment through corporate costs, technological innovation, industrial upgrading, and industrial transfer. To verify the effect of environmental regulation on social employment in different periods and under the intensity of environmental regulation, in this paper, environmental regulation is introduced as an influencing factor of social employment levels, based on China’s urban registration unemployment data from 1987 to 2017. A nonlinear smoothing autoregressive model is used to analyze the nonlinear long-term effect relationship between environmental regulation and social employment. The research results show that the relationship between environmental regulation and social employment does exhibit the characteristics of nonlinear transformation under different mechanisms, and the transformation speed is fast. The specific manifestation is that the environmental regulation has a restraining effect on social employment in the short term, and the environmental regulation has a promoting effect on social employment in the long term. Continued high-level environmental regulations will exacerbate the adverse impact of environmental regulations on social employment.

## 1. Introduction

Along with the slowdown of economic development and the fading of the demographic dividend in recent years, macroeconomic development under the ‘three-phase superposition’ has been facing the pressure of environmental protection and labor employment. According to the ‘13th Five-Year Plan’ toward national economic and social development, green development will be treated as the basic principle of China’s economic and social development during the ‘13th Five-Year’ period and even longer periods. As one of the primary methods to promote green development, environmental regulation is changing the track of economic growth by affecting the industrial restructuring and technological innovation, inevitably, having some impacts on social employment. Social employment refers to the activities of people with labor capacity and willingness to engage in various types of labor to obtain labor income; the workers provide goods and services to society through employment, while also obtaining labor income to provide material conditions for human survival. Social employment plays an important role in the sustainable development of society, and promotes the level of social employment, as one of the most essential goals of macroeconomic development, as well as the sustainable development of the Chinese economy. Overall, as one of the main factors influencing the social employment, what is the direction and intensity of the impacts of environmental regulation on employment? This paper intends to discuss the impact of environmental regulation on employment.

## 2. Literature Review

With the emergence of environmental issues and the slowdown of economic growth, the impact of environmental regulation on macroeconomic growth has become the main focus of researchers. In recent years, due to the increasing importance of environmental protection, more and more scholars have focused on exploring the impact of environmental regulation on employment, and different conclusions have been drawn due to the diversity of research perspectives. As such, domestic and foreign studies on the impact of environmental regulation on employment will be discussed, among which domestic scholars have conducted detailed studies on the relationship between environmental regulation and employment from the perspectives of industry heterogeneity, industrial upgrading, regional heterogeneity, geographical division, and urban–rural dual structure, respectively. When regard to social employment, this is an activity in which members of society engage in various types of labor to obtain legal labor remuneration or income. In this paper, the economic significance of social employment is taken into consideration, that is, laborers receive labor income while providing goods and services to society.

The views of foreign research are mainly divided into three categories. First, environmental regulation has a positive impact on employment. Roger H. Bezdek et al. (2008) found that environmental protection, economic growth, and employment creation are complementary and compatible based on environmental-related workload data at the US state level. Environmental protection creates jobs and replaces some employment, but overall the impact of environmental protection on employment is positive [1]. Second, environmental regulation has a negative effect on employment. Greenstone (2002) used the difference-in- difference (DID) method to show that the 1970 US Clean Air Act and the 1977 US Clean Air Act Amendment had a negative impact on employment; this law resulted in an decrease in GDP growth and an increase in social unemployment from 1973 to 1985 [2]. Third, the impact of environmental regulation on employment is uncertain. Jens Horbach and Klaus Rennings (2013) conducted surveys about community-level innovation from the perspective of enterprise and found that clean technology innovation can save costs and stimulate demand, thereby increasing employment, but water and air treatment technology innovations, dominated by high-end technology, have a negative impact on employment [3].

Recently, quite a number of domestic scholars assume that there is a U-shaped curve between environmental regulation and overall employment. With the increase of environmental regulation, its impact on employment has gradually turned positive [4,5,6,7], however, China’s environmental regulation and employment are currently in the decline stage of the U-shaped curve. If the employment structure is divided according to the pollution level and technical level, it will be found that the heterogeneity of the industry leads to significant differences in the shape and position of the U-shaped curve [5].

In heavily-polluting industries, there is a U-shaped relationship between environmental regulation and social employment. In moderate- and mildly-polluting industries, there is an inverted U-shaped relationship. The increase of labor cost share and the decrease of industrial monopoly degree will weaken the employment elasticity of environmental regulations [8]. For dirty industries, the impact of environmental performance on employment through technical effects is more significant, and thus, a win-win situation of ecological environment and employment stability will be achieved [9]. Environmental regulation has the strong endogeneity due to factors such as the employment level of the industry, the proportion of state-owned enterprises, the degree of foreign investment, and the amount of pollution emissions. Compared with the cleaning industry, environmental regulation has a greater impact on the employment of polluting industries [10]. Some scholars also assume that the correlation between environmental regulation and employment is not a simple U-shaped curve, but a complex nonlinear relationship, and that environmental regulation itself, industrial structures, and technological innovation have significant threshold effects [11].When environmental regulations are raised to a certain level, environmental regulation will promote employment in the industrial sector, and as the share of labor costs in the industrial sector rises, the impact of environmental regulation on employment will diminish [7].

From the perspective of industrial upgrading effect of environmental regulation, the implementation of environmental regulation policies alone will reduce the scale of regional employment and will not promote the employment of high-skilled labor. However, when considering the effect of industrial changes caused by environmental regulations, the industrial upgrading effect of environmental regulations promotes the growth of demand for high-skilled labor, although it does not bring the increase of employment scale. On the whole, the industrial transfer effect of environmental regulations promotes the increase of employment scale nationwide [12]. At present, coordination and matching between industrial environmental regulation and industrial structure adjustment have not been realized, so the interaction effect between environmental regulation and industrial structure rationalization cannot bring about employment promotion [13].

The heterogeneity of regional development is also an important factor affecting the relationship between the two. From the perspective of different labor income levels, due to the differences in industrial structure between regions, the employment effects of environmental regulations in different regions are also different; from the perspective of regions with different labor education levels, due to the regulation of enterprises and different levels of education labor, the effect is of a matching degree. The employment effect of environmental regulation in higher education and secondary education areas shows the effect of restraining and then increasing, and the environmental regulation in low education areas has a significant positive effect on employment [6]; divided by geographical area. From this perspective, the environmental regulations in the eastern region are more inclined to promote employment, while the central and western regions show a restraining effect on employment [7].

Considering the existence of the urban–rural dual employment structure, due to the ‘air blow effect’ and the difference in the employment positions of migrant workers with different levels of human capital, environmental regulation will increase the employment demand for high-skilled and low-skilled migrant workers and reduce the employment needs of middle-skilled migrant workers, resulting the ‘polarization’ phenomenon in employment; with the gradual decline of the labor market segmentation, environmental regulation has changed from the perpetual effect on migrant workers to the promotion effect. In general, the negative impact of environmental regulation on the employment of urban migrant workers is greater than the employment of urban local labor [14,15,16].

According to the above research, most scholars believe that there is a positive, negative, or U-shaped curve relationship between environmental regulation and employment, ignoring the dynamic relationship between environmental regulation and employment, that is, the linear relationship between environmental regulation and employment.

## 3. Analysis of the Impact Mechanism of Environmental Regulation on Employment Level

This article believes that environmental regulation can affect social employment through various paths such as production costs, technological innovation, and industrial upgrades and transfers. However, considering that the completion of technological innovation by enterprises or the completion of industrial transformation and upgrading are all gradual processes, the impact of environmental regulation on social employment is long-term. At the same time, the impact of environmental regulation on different paths varies, so the impact of environmental regulation on social employment is nonlinear. The main innovations of this paper are as follows: Previous studies focusing on the impact of economic development on employment, this paper studies the impact of environmental regulations on social employment, and the smooth transition autoregressive (STR) model is used to analyze environmental regulations as well as the nonlinear relationship between residents’ employment. This paper can enrich relevant research in the field of environmental protection and employment. Among the impacts, the direct effect is mainly caused by the cost effect of environmental regulation, which changes the level of social employment, and the indirect effect is mainly caused by the technological innovation effect, industrial upgrading effect, and industrial transfer effect produced by environmental regulation.

From the perspective of the cost effect of environmental regulation [17,18,19], ‘follow cost theory’ holds the idea that the government adopts preferential tax policies and market discipline-type environmental regulation means, such as administrative penalty tax on polluting industries, which will increase the three highs’ industry’s operating costs, and inhibit the scale expansion and market competitiveness of polluting industries, thus, causing the collapse of some enterprises due to unable to bear the cost pressure. Enterprises that intend to enter the polluting industry will also be rejected because of the high threshold of environmental regulation, which will reduce the employment space of high-polluting industries. The generation of cost pressure will also have the substitution effect between the pollution factor and the labor factor. When the cost pressure causes the price of the pollution factor to be higher than the labor factor price, the labor factor replaces the pollution element in the production market. At the same time, the government reduces the operating costs of green environmental protection enterprises through tax subsidies and other preferential policies and promotes green environmental protection enterprises to expand production scale and enhance their market competitiveness.

From the perspective of the technological innovation effect of environmental regulation [20,21,22], the ‘innovation compensation theory’ holds that environmental regulation will encourage enterprises to carry out technological innovation in order to take the leading role in market competition. Under the pressure of environmental regulation, some enterprises realize that the effect of innovation compensation can enhance their competitive advantages, improve their production efficiency, expand their business scale, and enhance their employability. However, some enterprises may suffer large external shocks when facing the environmental regulation due to the fact that they fail to take advantage in the field of technological innovation of enterprises, as well as some other factors, such as insufficiency operating ability, weak financing ability, and poor innovation ability. In this way, the scale of production will shrink and even the enterprise itself will be eliminated out of the market as a result of the losses of labour force and the transfer of labour force. Judging from the effects of the current environmental regulation policies, environmental regulation policies have not provided sufficient motivation for green technological innovation [23].

From the point of view of the industrial upgrading effect of environmental regulation [24,25,26], industrial upgrading can be divided into the improvement of industrial quality and efficiency caused by technological upgrading and can also be reflected in the adjustment and improvement of industrial structure. Environmental regulation encourages enterprises to upgrade their technology, thereby improving the efficiency of the industry, increasing the demand for highly skilled labor, and reducing the demand for low-skilled labor, causing a substitution effect of high-skilled labor for low-skilled labor. At the same time, heavy industries relying on resource consumption in the early stages of industrialization can no longer be the pillar of economic growth, and the industrial structure is transforming into high-tech industries, high-end manufacturing, and service industries, with the continuous improvement of environmental regulation. The change of industrial structure has reduced the number of labor positions in the polluting industry, while the employment opportunities of new environmentally-friendly industries and service industries increased.

From the perspective of industrial transfer effect of environmental regulation [27,28,29], the eastern region has taken the lead in entering the stage of rapid development under the background of reform and opening-up. In addition, the eastern region has accumulated a solid material foundation with the rough and mad economic development mode. Compared with the central and western regions, the pillar industries of economic development in the eastern region seem to be more diversified, and the high-tech industries driven by innovation in the eastern region are relatively denser. On the contrary, the development of the central and western regions started relatively later, and the intensity of environmental regulation was relatively weak, thus, providing some opportunities and chances for the polluting industry to continue to survive and develop. When the cost of local upgrading in a region with strict environmental regulation is higher than the cost of regional transfer, environmental regulation will lead to the transfer of enterprises from a more environmentally regulated area to a more relaxed area of environmental regulation. The transfer of industries promotes the industrialization process for the transfer areas, and the transfer of the corresponding supporting industries also brings a large number of employment opportunities to the transfer areas. For the industrial relocation, the ‘three high’ industry’s move out and bankruptcy leads a series of results, such as the large loss of employment and the increase of unemployment rate. In addition, the transformation and upgrading of the industry in the emigration area force the enterprises to expand the investment in technology and capital as well as the demands for high-tech labor.

## 4. Empirical Analysis

### 4.1. Construction of a Measurement Model

In view of the complex relationship between environmental regulation and employment, the smooth transition regression model (STR) is taken into consideration to help the analysis. The STR model is a typical nonlinear model, which was first proposed by Granger and Terasvirta to describe the transition from one mechanism to another [30]. The smooth transition regression model is based on the linear model, and further developed according to the mechanism transformation theory, which is mainly used to describe the transition smoothing relationship between the two extreme mechanisms to explain the relationship and law between different economic phenomena.

The standard form of the smooth transition regression (STR) model is as follows [31,32,33]:

(1) $$y_{t}={\phi^{\prime }}^{zt}+{\theta^{\prime }}^{zt}G(\gamma ,c,s_{t})+\mu_{t},\;t=1,\ldots ,T$$

(2) $$G(\gamma ,c,s_{t})={\left\{1+\exp\left[-\gamma \prod_{k=1}^{k}s_{t}-c_{k}\right]\right\}}^{-1},\gamma >0$$

(3) $$G(\gamma ,c,s_{t})=1-\exp\left[-\gamma {\left(s_{t}-c\right)}^{2}\right],\gamma >0$$

$y_{t}$ is the dependent variable, which is denoted by the urban registered unemployment rate in China. $z_{t}$ is the explanatory variable vector, which is a social factor that may affect the explanatory variables, where $z_{t}$ = ($w_{t}^{\prime },x_{t}^{\prime }$)′, and $w_{t}^{\prime }=\left(1,y_{t-1},\ldots ,y_{t-p}\right)$′ are lag p-order variable that interprets the variable $y_{t}$, and $x_{t}^{\prime }$ = ($x_{1t},\ldots ,x_{kt}$)′ are the lag variables of other exogenous variables. $\phi =(\phi_{0},\phi_{1},\ldots \phi_{p})$ and $\theta =(\theta_{0},\theta_{1},\ldots \theta_{m})$ are the linear and nonlinear parameter vectors of the STR model, respectively. The transformation function $G(\gamma ,c,s_{t})$ is a continuous function between 0–1, and the function value relies on $\gamma ,c,s_{t}$, while, $s_{t}$ is a conversion variable, which can be either the part of $z_{t}$, or an exogenous variable not included in $z_{t}$. $\mu_{t}$ is an independent and identically distributed error sequence term. $c=(c_{1},c_{2},\ldots c_{k})$ represents the time or position of the state transition, which is the threshold value under different mechanisms. The smoothing parameter γ indicates that the speed converted from one mechanism to another when the interpreted variable is under the influence of the conversion variable. The transformation function $G(\gamma ,c,s_{t})$ in the STR model usually has two forms. When G is present the same form as in Equation (2), it corresponds to the logistic smooth transition autoregressive (LSTR) model, and when G is in the same form as Equation (3), it corresponds to the exponential smooth transition autoregressive (ESTR) model.

The K value in the LSTR model is usually 1 or 2 [31]. When K=1, the transfer function G is an odd function and is a monotonically increasing function of the conversion variable $s_{t}$, that is the LSTR1 model. Its general function form is:

(4) $$G(\gamma ,c,s_{t})={\left\{1+\exp\left[-\gamma (s_{t}-c_{1})\right]\right\}}^{-1},\;\gamma >0$$

when K=2, the transfer function G does not have monotonicity, and is symmetric about $\left[\frac{c_{1}+c_{2}}{2},G(\frac{c_{1}+c_{2}}{2})\right]$, and when $s_{t}=\frac{c_{1}+c_{2}}{2}$, the function G takes the minimum value, that is the LSTR2 model.

(5) $$G(\gamma ,c,s_{t})={\left\{1+\exp\left[-\gamma (s_{t}-c_{1})(s_{t}-c_{2})\right]\right\}}^{-1},\gamma >0,c_{1}\leq c_{2}$$

### 4.2. Data Sources and Explanations

This paper selects the social employment level as the explanatory variable, denoted by the urban registered unemployment rate (EMP). The environmental regulation is selected as the core explanatory variable and is expressed by the environmental regulation intensity index (ER), and the ER is evaluated from the two perspectives including the environmental governance cost and governance performance. Environmental governance cost is measured by the two indexes; one is denoted by the proportion of the industrial pollution control investment to the industrial added value and the other one is denoted by the proportion of sewage charge collection amounts to industrial pollution. The governance performance is measured by the comprehensive utilization rate of industrial waste. Finally, under the help of the entropy weight method, the weight of each indicator of environmental regulation is calculated one by one, and the weighted value is considered as the environmental regulation intensity index.

Considering that there are many factors influencing the level of social employment, the economic development level (RJGDP) and the industrial structure upgrade (CY) are selected as the control variables. The economic development level is measured by GDP per capita, and the level of the industrial structure upgrading is evaluated by the proportion of the tertiary industry’s output value to the total output value. Logarithms for the above variables were taken into consideration to reduce the occurrence of heteroscedasticity in the empirical analysis. The data of environmental regulation index, social employment level, and other economic data are all from the China Environmental Yearbook, China Environmental Statistics Yearbook, and China Statistical Yearbook. The empirical process is mainly implemented by the JMulTi software.

### 4.3. The Empirical Process

#### 4.3.1. Stationarity Test

The smoothness of the data is necessary for nonlinear testing and estimation. It can be seen from Table 1 that there is no unit root after the first-order difference between lnEMP and lnER, which is a stable time series. The cointegration test requires that the variables should be the same order and single, so there are EMP~(1), ER~(1). The co-integration test is performed on EMP and ER, and the test results are shown in Table 2 below, which show the long-term cointegration relationship between environmental regulation and unemployment, and that this relationship is stable. The co-integration equation is as follows:

(6) ΔlnEMP−0.134697ΔlnER=0

(7) ΔlnEMP=0.134697ΔlnER

According to the above co-integration equation, it can be shown that there is a positive correlation between environmental regulation and unemployment rate, which means that the strengthening of environmental regulation will reduce the level of social employment. It is economically consistent with China’s current stage of economic development, that is; most highly polluting and energy-consuming enterprises have been closed under the implementation of current environmental regulations, while new industries have not yet formed, and the ability to absorb employment is limited. In the sample interval, there is a positive correlation between environmental regulations and unemployment.

#### 4.3.2. Determination of the Lag Order

When environmental regulation returns to the level of social employment, it is first necessary to judge whether environmental regulation has significant nonlinear transformation characteristics for social employment. The AIC and SC criteria in the vector autoregressive model (VAR) are used to select the lag order, and the lag order of the AR part is chosen to be two orders in here. The basic form of the model is as follows:

(8) $$\begin{array}{c} \Delta \ln EMP=0.0064+0.166\Delta \ln EMP(-1)-0.250914\Delta \ln EMP(-2)+0.009\Delta \ln ER\\ \left(0.168647)\;(1.145473)\;(-1.738710)\;(0.696597\right)\\ +0.013\Delta \ln ER(-1)+0.100\Delta \ln RJGDP-0.187\Delta \ln CY\;(1.083282)\;(0.421269)\;(-0.531677)\\ R^{2}=0.251213,\;AIC=-2.894279,\;SC=-2.561228,\;\mathrm{DW}=1.181019 \end{array}$$

After the adjustment, the model has low goodness-of-fit, but the fitting effect is not ideal. Therefore, it is tested whether there is a nonlinear relationship between environmental regulation and the employment level of residents, and whether the fitting effect of the model will be greatly improved after the transformation becomes a nonlinear relationship.

According to the test results in Table 3, when the conversion variable is ΔlnER*, the probability of accepting the linear relationship hypothesis is 4.5991 × 10^−3^, which is less than 5%, so the hypothesis about the linear relationship between environmental regulation and employment level can be rejected. The alternative hypothesis, that there is a nonlinear relationship between the two factors, should be taken into consideration. Since the *p* value of F3 is the smallest among F4, F3, and F2, the corresponding form of the conversion function is LSTR2.

#### 4.3.3. Determination of Initial Values of Smoothing Parameters and Positional Parameters

**Figure 1:**
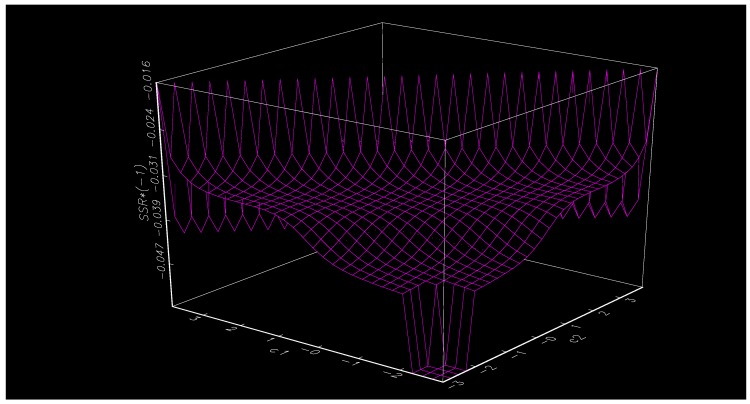
Plan of the grid search.

**Figure 2:**
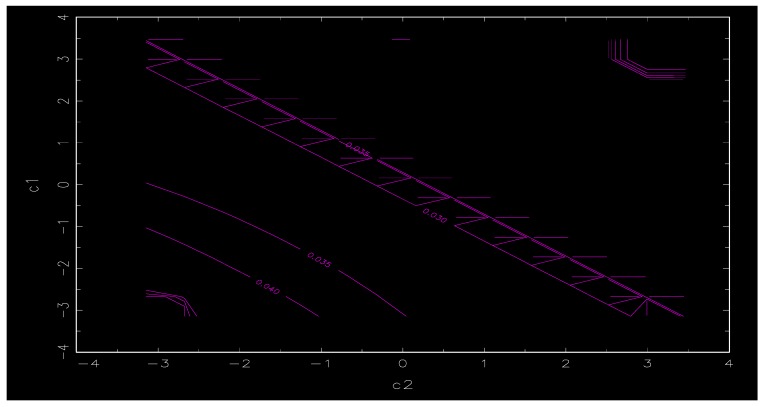
Contour map of grid search.

It is necessary to estimate the parameters of the STR model after the determination of the conversion form of the function and the conversion variables. According to the systematic grid search method, the chosing of the initial estimate of c (location parameter) and γ (smoothing parameter) are performed by selecting different γ and c within a certain range so that the sum of squared residuals estimated by the STR model system is the smallest. As shown in Table 4, the interval of the smoothing parameter γ is set to be (0.50, 10), and the interval of the positional parameter c is (−3.38, 3.47) (the smoothing parameter interval and the position parameter interval are set based on the model system data change and the conversion variable empirical data range, respectively), and the value of γ and c are both 30, which constitutes a combined point of 30 × 30 (γ,c). All the two-dimensional space combination points are evaluated one-by-one to find the parameters with the smallest residual square sum as the initial estimate value for further optimization. The initial estimates of γ and c are shown in the table below. Figure 1 and Figure 2 are contour plots and plans, respectively, under the help of two-dimensional grid search method, and the plan is the inverse of the maximize residuals.

#### 4.3.4. Determination of Model Parameters

The Newton–Paphson method is used to solve the maximum conditional relief function after the determination of the parameters and initial variables. The nonlinear equation parameters φ, θ, γ, and c for environmental regulation and employment levels can be obtained. Detailed results are shown in Table 5:

The specific form of the LSTR2 model is as follows:

(9) $$\begin{array}{c} \Delta \ln EMP=-0.86499-18.48528\Delta \ln EMP(-1)+7.56604\Delta \ln EMP(-2)+1.5224\Delta \ln ER\\ -1.08339\Delta \ln ER(-1)-5.79462\Delta \ln RJGDP-3.86579\Delta \ln CY\\ +G(\gamma ,c,\Delta \ln ER)\\ \times [1.85518+42.47549\Delta \ln EMP(-1)-17.36590\Delta \ln EMP(-2)-0.78558\Delta \ln ER\\ +2.50626\Delta \ln ER(-1)+13.78695\Delta \ln RJGDP\\ +8.83928\Delta \ln CY] \end{array}$$

(10) $$\begin{array}{c} G(\gamma ,c,\Delta \ln ER)={\left\{1+\exp\left[10.32112(\Delta \ln ER-0.08791)(\Delta \ln ER+0.21572)\right]\right\}}^{-1}\\ R^{2}=0.796,AIC=-6.3193,SC=-5.5105 \end{array}$$

According to the above results, the employment effect of environmental regulation shows a clear transition relationship. This shows the better performance of the LSTR2 model, that is to say, the model can better demonstrate the nonlinear relationship between environmental regulation and employment level, and the estimated coefficient of the model has strong significance.

The nonlinear part obtains the positional parameters $c_{1}=-0.08791$ and $c_{2}=0.21572$, and the transfer function is $\frac{c_{1}+c_{2}}{2}=0.063905$. Therefore, when the conversion variable ΔlnER=0.063905, the transfer function is G=0, that is, the nonlinear portion does not exist. The model then only presents as the linear part:

(11) $$\begin{array}{c} \Delta \ln EMP=-0.86499-18.48528\Delta \ln EMP(-1)+7.56604\Delta \ln EMP(-2)+1.52240\Delta \ln ER\\ -1.08339\Delta \ln ER(-1)-5.79462\Delta \ln RJGDP-3.86579\Delta \ln CY \end{array}$$

When it comes to the linear part of the model, it is clear that the unemployment rate coefficient is negative, and the government will respond to the current employment problem by taking out the corresponding employment policy in the next year, thereby, the unemployment rate may have a reduction. The policy can solve the unemployment problem in the short-term, but in the long run, the unemployment problem caused by the economic operation has a cumulative effect, so the coefficient of the unemployment rate lags behind the second period is positive. There is a positive correlation between Δln*ER* and unemployment rate, with a coefficient of 1.52240, and it is tested at a significance level of 1%, which proves that the implementation of environmental regulation has a loss effect on social employment in the current period. The relationship between ΔlnER(−1) and unemployment rate is negative at a significance level of 1%, the result indicates that the environmental regulation lags behind the first phase has an expansion effect on social employment. By the way of technological innovation and the adjustment of industrial structures, such as paths to reduce the unemployment rate, namely, environmental regulation has a positive effect on employment for a long time. There is a negative correlation between per capita GDP and industrial structure upgrading and unemployment rate. That is, economic development and upgrading of industrial structure can effectively reduce unemployment and promote the improvement of social employment levels. The implementation of environmental regulations will also have a positive impact on economic growth, thereby promoting the level of social employment [34].

When the conversion variable Δln*ER* is equal to the critical value, that is, Δln*ER* = −0.08791 or Δln*ER* = 0.21572, the transfer function G = 1/2, and the model is in the transition state from the pure linear state to the nonlinear model. The basic form of the model is:

(12) $$\begin{array}{c} \Delta \ln EMP=0.0626+2.752465\Delta \ln EMP(-1)-1.11655\Delta \ln EMP(-2)+1.12946\Delta \ln ER\\ +0.16974\Delta \ln ER(-1)+1.098855\Delta \ln RJGDP+0.55388\Delta \ln CY \end{array}$$

When switching variable Δ ln*ER* < −0.08791 or Δ ln*ER* > 0.21572; namely, the intensity of environmental regulation is decreased, and the speed of decrease is more than 8.41% [exp(0.08791) −1]; or when the intensity of environmental regulation is rapidly increased, and the speed exceeds 24.07% [exp(0.21572) −1], the nonlinear effects of environmental regulation on employment will change significantly. Then, the basic form of the model is:

(13) $$\begin{array}{c} \Delta \ln EMP=-0.86499-18.48528\Delta \ln EMP(-1)+7.56604\Delta \ln EMP(-2)+1.52240\Delta \ln ER\\ -1.08339\Delta \ln ER(-1)-5.79462\Delta \ln RJGDP-3.86579\Delta \ln CY\\ +G(\gamma ,c,\Delta \ln ER)\\ \times [1.85518+42.47549\Delta \ln EMP(-1)-17.36590\Delta \ln EMP(-2)-0.78558\Delta \ln ER\\ +2.50626\Delta \ln ER(-1)+13.78695\Delta \ln RJGDP\\ +8.83928\Delta \ln CY] \end{array}$$

When the conversion variable is satisfied with −0.08791<ΔlnER<0.21572, that is the slow process of environmental regulation, the transfer function value is small, and the conversion variable ΔlnER has small impact on the entire nonlinear part, environmental regulation and employment level (unemployment rate) will maintain the linear relationship. That is to say that environmental regulation will have a negative effect on the unemployment rate with the coefficient of 1.52240, indicating that environmental regulation will increase the unemployment rate, which is not conducive to the improvement of social employment level. The main reason is that the implementation of environmental regulation in the short term will have a phase-out effect on some ‘three high’ enterprises, and the green environmental protection industry in the incubation has not yet formed, resulting in the loss of social employment.

The smoothing parameter of the model is γ=10.32112 (it is generally considered that the adjustment speed of the nonlinear part is faster when γ>10), indicating that the adjustment speed of the nonlinear part of the model is relatively faster, the conversion function G is an increasing function of the conversion variable ΔlnER, and the conversion function grows as the value of the variable grows, thus, the nonlinear part in the model has a greater influence on the level of social employment.

**Figure 3:**
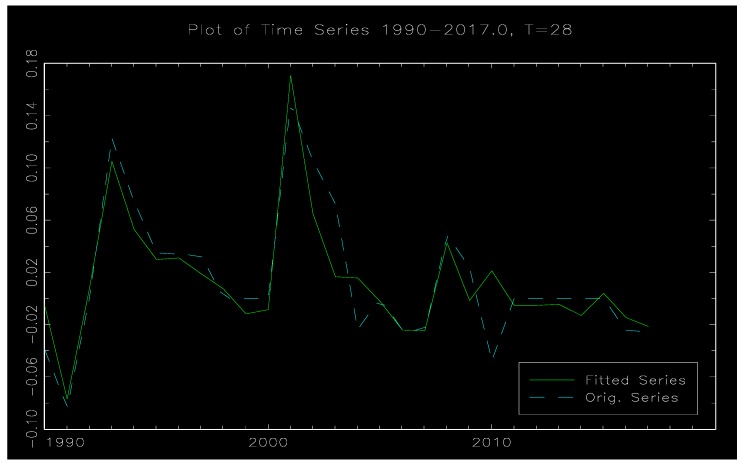
Original and fitted data time series diagram.

Figure 3 is a time series diagram of the raw data and simulation data in the model. It can be seen from the following figure that the dynamic characteristics of the data fitted by the STR model have a high degree of coincidence with the dynamic characteristics of the original data, which indicates the effectiveness of the STR model. That is to say, the model can better fit the dynamic relationship between environmental regulation and employment.

**Figure 4:**
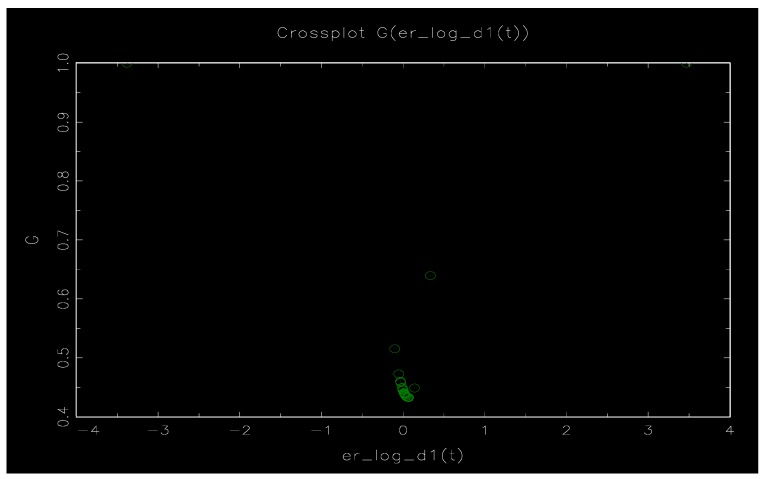
Schematic diagram of the conversion function G.

**Figure 5:**
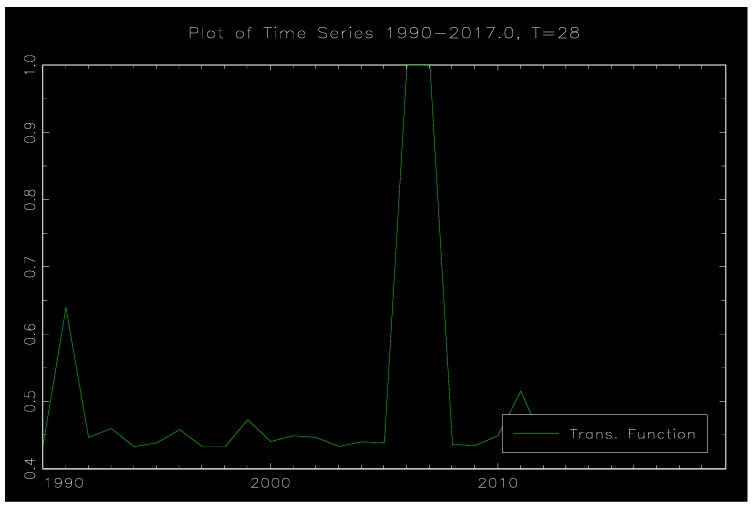
Time series diagram of the zone conversion.

Figure 4 and Figure 5 show schematic diagrams of the model nonlinear function and the transfer function G(γ,c,ΔlnER). Figure 4 is the result of the transfer function in which ΔlnER is treated as the conversion variable. The horizontal axis represents the conversion variable ΔlnER and the vertical axis represents the conversion function G. It can be seen that the value interval of the conversion function G is 0–1, and the symmetry about ΔlnER=0.063905. Figure 5 is the time series diagram of the transfer function, and it clearly shows there are obvious phase characteristics between environmental regulation and social employment. Specifically, it can be divided into three stages: 1990–2004, 2005–2008, and 2009–present. Among them, the values of G in the two periods from 1990 to 2004 and 2009 to present are relatively small and show the linear performance. The lagging period of environmental regulation is negatively correlated with the unemployment rate, which indicates that such environmental regulation is conducive to the improvement of social employment in the long run. In 2005–2008, during the ‘Eleventh Five-Year Plan’ period, there was an obvious nonlinear characteristic between environmental regulation and social employment (G=1). At this time, the coefficient of ΔlnER is 0.73682, and the coefficient of ΔlnER(−1) is 1.42287. The coefficient of the environmental regulation lags from the first phase is changed from the negative value of the linear part (such as Equation (9)) to the positive value, demonstrating the positive correlation between the environmental regulation lag phase 1 and the social unemployment rate, which is unfavorable to the improvement of the social employment level. We assume that the change in the coefficient is mainly affected by the domestic environmental protection situation. The ‘Eleventh Five-Year Plan’ is the period in which China’s environmental protection situation has taken turns, and the ‘environmental storm’ has become the key word during the ‘Eleventh Five-Year Plan’ period. The five-year environmental plan is considered to be the best environmental plan for the past years at the government work evaluation meeting. During the ‘Eleventh Five-Year Plan’ period, the Ministry of Environmental Protection will not accept and approve the investment of more than 2.9 trillion yuan for 813 projects that do not meet the environmental protection requirements. At the same time, it will investigate and deal with heavy metal pollution, papermaking enterprises, sewage treatment plants, etc., and shut down more than 20,000 illegal sewage companies. The long-term and super-level improvement of environmental regulation has led to a large loss of social employment. Therefore, during the period, whether it is the current period of environmental regulation or the first period of lag, there is a significant positive correlation between the unemployment rate.

#### 4.3.5. Model Stationarity Test

In order to evaluate the stability of the model, the ADF and Philliips& Perron (PP) unit root test method is used to test the stability of the residual term of the regression model. The results of the test are shown in Table 6. The residual term of the regression model is a stable time series at a significance level of 5%, whether under the ADF test or the PP test.

The ARCH-LM test method is used to test the heteroscedasticity of the model. The corresponding results are shown in Table 7. The chi-square statistic is 1.4168, the corresponding *p* value is 0.4924, and the F-statistic is 0.7492, the corresponding *p* value is 0.4839. The null hypothesis is accepted at a significance level of 10%, that is, there is no heteroscedasticity in the residual term.

The results show that the impact of environmental regulation on the rate of unemployment presents a nonlinear conversion relationship according to different intensities of environmental regulation. In the short period, the correlation between environmental regulation and unemployment rate is positive, and environmental regulation will cause the loss of employment; in the long-term, the correlation between environmental regulation and unemployment rate is negative, and environmental regulation will cause the expansion of employment. At the same time, the long-term and high-intensity environmental regulation will contribute to the loss of social employment. In particular, long-term implementation of high-intensity environmental regulation policies will have a major influence on social employment, resulting in a significant reduction in employment.

## 5. Discussion

According to the research on the mechanism of the impact of environmental regulation on the employment of residents, this paper revealed that, from the perspective of the effects of cost, technological innovation, industrial upgrading, and industrial transfer, that the correlation between environmental regulation and employment is neither immutable nor a U-shaped relationship. Rather, the effects of environmental regulation on employment depends on the intensities of change on different periods of time. To verify this correlation, this paper used the STR nonlinear model to study the correlation between environmental regulation and social unemployment rates in China from 1987 to 2017. Studies have shown that the transition of nonlinear conduction of environmental regulation to social employment between different mechanisms appears smooth and continuous, and the transformation speed is accelerated.

Environmental regulation is a factor that affects social employment. Environmental regulation will have a negative impact on the current levels of social employment. A lag in environmental regulation will have a positive impact on the current levels of social employment. With the sharp increase in the intensity of environmental regulations, the impact of lagging environmental regulations on the level of social employment has also changed from positive to negative. This result is strong proof of the negative impact of environmental regulations on China’s employment levels at present, especially the high-intensity environmental regulations during the ‘Eleventh Five-Year Plan’ period, which have created long-term job losses. However, this loss is only temporary. In the long run, the impact of environmental regulations on social employment is still positive.

The coordinated development of environmental protection and social economy is an important issue for various countries in the world, especially for the developing countries to achieve sustainable economic and environmental development. In this work, it can be seen that environmental regulation will not adversely affect social employment in the long run in China’s practice, but will improve the level of social employment, indicates that environmental protection and green development are in line with the trend of economic and social development. Meanwhile, it is also found that the intensity of environmental regulations and economic development should be coordinated with each other. Otherwise, once the environmental regulations extend beyond the bearing capacity of social development in a certain period of time, the high-intensity environmental regulations will lead to the loss of social employment in a short time. Therefore, it is also necessary for other countries to implement environmental regulations in the process of economic development. However, the intensity of environmental regulations in each period should be adapted in accordance with the affordability of their own economic development. Economic development and social employment are especially indispensable in developing countries. Especially, environmental protection should be regarded as an important part in the process of economic development. Economies should be developed with the goal of protecting environment, as environmental protection helps to improve the development of economic and the level of social employment continuously. The two are interconnected and mutually reinforcing. Specifically, different countries and regions have different economic development stages and environmental conditions. As such, the following three aspects may be taken into consideration to help achieve the coordinated development of environmental regulations and social employment. First, improve the system design of environmental regulations. The use of public goods is inseparable from the government’s macro-control. As the coordinator of environmental protection and economic development, the government should make full use of its role in environmental protection. Second, the market should play a fundamental role in environmental protection. The profitability of the market and the public properties of environmental goods are not contradictory. When the environment weakens the profitability of an industry or a company, the public properties of the environmental goods will also diminish. Third, some supports should be given to innovations and entrepreneurships related to environmental protection.

## Figures and Tables

**Table 1:** Augmented Dickey-Fuller test (ADF) stationarity test results.

Variable	T Statistic	10% Threshold	DW	AIC	SC	Inspection Form
lnEMP	−1.331972	−3.218382	1.838162	−2.554949	−2.414829	(C,T,2)
ΔlnEMP	−5.855128	−3.225334	1.187449	−3.049996	−2.859682	(C,T,2)
lnER	−5.581004	−3.218382	2.015744	2.031232	2.171352	(C,T,1)
ΔlnER	−8.778255	−3.221728	2.321160	2.557491	2.698935	(C,T,1)
lnRJGDP	−2.668360	−3.221728	1.331849	−3.424563	−3.235971	(C,T,1)
ΔlnRJGDP	−3.540273	−3.225334	2.019817	−3.566482	−3.376167	(C,T,1)
lnCY	−2.492138	−3.221728	2.224492	−3.583519	−3.394926	(C,T,1)
ΔlnCY	−3.476764	−3.221728	1.862562	−3.430597	−3.286299	(C,T,1)

**Table 2:** Johansen cointegration test.

**a. Unrestricted cointegration rank test (trace).**				
**Null Hypothesis**	**Eigenvalues**	**Trace Statistic**	**5% Significant Level of Critical Value**	***p* Value**
None	0.637587	49.07211	15.49471	0.0000
At most 1	0.521741	20.65289	3.841466	0.0000
**b. Unrestricted cointegration rank test (maximum eigenvalue).**				
**Null Hypothesis**	**Eigenvalues**	**Maximum Eigenvalue Statistic**	**5% Significant Level of Critical Value**	***p* Value**
None	0.637587	58.41922	14.26460	0.0002
At most 1	0.521741	20.65289	3.841466	0.0000

**Table 3:** Conversion function test selection results.

Conversion Variable	F1	F4	F3	F2	Model Form
ΔlnEMP(−1)	NaN	NaN	0.81322	0.38642	Linear
ΔlnEMP(−2)	NaN	NaN	0.33050	0.67987	Linear
ΔlnER *	4.5991 × 10^−3^	3.5129 × 10^−2^	1.1601 × 10^−3^	0.47002	LSTR2 *
ΔlnER(−1)	0.39623	0.38287	0.27134	0.69531	Linear
Trend	NaN	NaN	0.29075	0.24284	Linear

Note: F1, F4, F3, and F2 represent F statistics, respectively, and * represents the form of the optimal transition variable and conversion function determined by the STR model.

**Table 4:** Initial estimation results of smoothing parameters and positional parameters.

Sum of Residuals	Smoothing Parameter γ	Interval	Position Parameter c_1_	Position Parameter c_2_	Interval
0.0158	10.0000	(0.50, 10.00)	−0.0742	0.1620	(−3.38, 3.47)

**Table 5:** LSTR2 model parameter estimation results.

	Variable	Initial Value	Estimated Value	Standard Deviation	t Statistic	*p* Value
Linear part	CONST	0.32049	−0.86499	0.0000	0.0000	0.0350
	ΔlnRJGDP	−16.25790	−5.79462	0.0000	0.0000	0.0097
	ΔlnCY	−9.73319	−3.86579	10.1023	−0.3827	0.7093
	ΔlnEMP(−1)	−31.93346	−18.48528	0.0000	0.0000	0.0757
	ΔlnEMP(−2)	7.10019	7.56604	0.0000	0.0000	0.0133
	ΔlnER	0.65831	1.52240	0.4543	3.3514	0.0065
	ΔlnER(−1)	−1.45708	−1.08339	0.0000	0.0000	0.0002
Nonlinear part	CONST	−0.74772	1.85518	0.0000	0.0000	0.1492
	ΔlnRJGDP	35.42354	13.78695	0.0000	0.0000	0.1229
	ΔlnCY	20.88360	8.83928	23.1534	0.3818	0.7099
	ΔlnEMP(−1)	69.32922	42.47549	0.0000	0.0000	0.0454
	ΔlnEMP(−2)	−15.44381	−17.36590	0.0000	−0.0000	0.0368
	ΔlnER	0.15577	−0.78558	0.3778	−2.0792	0.0618
	ΔlnER(−1)	3.18715	2.50626	0.0000	0.0000	0.0000
	γ	10.00000	10.32112	0.0000	0.0000	0.0306
	$c_{1}$	−0.07416	−0.08791	0.0437	−2.0105	0.0695
	$c_{2}$	0.16205	0.21572	0.0000	0.0000	0.0167
AIC −6.3193	SC −5.5105	HQ −6.0721	$R^{2}$ 0.7962	${\bar{R}}^{2}$ 0.8035	SSR 0.0014	SDR 0.0369

**Table 6:** Residual stability test.

Testing Method	T Statistic	10% Threshold	*p* Value	Conclusion
ADF test	−4.013825	−3.229230	0.0205	smooth
PP test	−4.037300	−3.229230	0.0195	smooth

**Table 7:** ARCH-LM test (with two lags).

Chi-Square Statistic	1.4168	*p* Value	0.4924
F statistic	0.7492	*p* Value	0.4839
